# A Monte Carlo Study of Knots in Long Double-Stranded DNA Chains

**DOI:** 10.1371/journal.pcbi.1005029

**Published:** 2016-09-15

**Authors:** Florian C. Rieger, Peter Virnau

**Affiliations:** 1 Institute of Physics, Johannes Gutenberg University Mainz, Mainz, Germany; 2 Graduate School Materials Science in Mainz, Mainz, Germany; Florida State University, UNITED STATES

## Abstract

We determine knotting probabilities and typical sizes of knots in double-stranded DNA for chains of up to half a million base pairs with computer simulations of a coarse-grained bead-stick model: Single trefoil knots and composite knots which include at least one trefoil as a prime factor are shown to be common in DNA chains exceeding 250,000 base pairs, assuming physiologically relevant salt conditions. The analysis is motivated by the emergence of DNA nanopore sequencing technology, as knots are a potential cause of erroneous nucleotide reads in nanopore sequencing devices and may severely limit read lengths in the foreseeable future. Even though our coarse-grained model is only based on experimental knotting probabilities of short DNA strands, it reproduces the correct persistence length of DNA. This indicates that knots are not only a fine gauge for structural properties, but a promising tool for the design of polymer models.

## Author Summary

We develop a coarse-grained model of double-stranded DNA which is solely based on experimentally determined knotting probabilities of short DNA strands. Our analysis is motivated by the emergence of DNA nanopore sequencing technology. The main advantage of nanopore sequencing in comparison to next-generation devices is its capability to sequence rather long DNA strands in a single run, currently up to ≈10,000 base pairs. Unfortunately, long DNA strands easily self-entangle into knotted conformations, and sequencing knotted DNA with nanopores may be subject to error. In our manuscript, the typical extent and likelihood of DNA knots is computed for DNA chains of up to half a million base pairs, and we estimate the abundance of complex and composite knots in relation to DNA length. Our analysis indicates that DNA knots may be a formidable roadblock for the development of devices which support substantially longer read lengths. We also show that structural properties of DNA, like its resistance to bending, are intimately linked to the molecule's tendency to form knots. We demonstrate how this connection can be utilized to introduce mathematical models of DNA which account for the molecule's overall statistical properties.

## Introduction

Entanglements in molecular cords like polymers or semi-flexible biopolymers like double-stranded DNA (dsDNA) often lead to knotted chain conformations. The significance of DNA knots has been discussed in biological contexts [1, 2, 3], as well as in technological settings: Recent studies [4, 5] investigated how knots in DNA change the translocation of long DNA molecules through nanopores. DNA translocation dynamics are of practical importance in the context of DNA nanopore sequencing, where a single molecule of either single- or double-stranded DNA is electrophoretically driven through a nano-scale pore across an impermeable thin membrane. The DNA's translocation through the nanopore alters the amplitude of the electrochemical current by perturbing the charge transport along the pore [6, 7]. In a common approach, the chain's nucleotide sequence is read by directly analysing the time-dependence of the current signal [8].

Mathematically, knots are only well-defined in closed curves. Nevertheless, a physical definition is often applied to open strings [9, 10]: Ends are connected in a systematic manner before the knot type is analysed (see the Methods section). Knots are categorised by their minimal number of crossings in planar projection. The simplest (non-trivial) knot is the so-called trefoil, which has three crossings and can be obtained by closing an overhand knot. Intriguingly, some of our intuitive understanding of macroscopic knots carries over to the nano-scale. About 50 years ago, Frisch and Wasserman conjectured [11] that any molecular cord will eventually be knotted as the chain length increases. This conjecture was later proven for certain classes of lattice polygons [12], but does not state how polymer length and polymer properties are related to knotting probability. Polymers in globular states [13–15, 9] and DNA in viral capsids [16, 17, 3, 18] are known to be highly knotted, whereas unconstrained polymers [15, 19, 9] or DNA in good solvent conditions are less prone to self-entanglements. The probability of knotting in dsDNA was first measured in the early 1990s by gel electrophoresis [20, 21]. Strand sizes of up to ≈10,000 base pairs were considered and depending on salt conditions, the probability of observing knots was at most a few percent. Consequently, self-entanglements and knots have mostly been ignored in the context of nanopore sequencing. Reference [20] also describes for the first time a coarse-grained model for DNA which is partly based on knotting probabilities. Very recently, a ground breaking study (Plesa *et al*., Nature Nanotechnology in press) has pushed these boundaries even further and estimated knotting probabilities for significantly larger strands in high salt concentrations by analysing translocation events in solid-state nanopores.

The focus of this work is two-fold. First, we introduce a coarse-grained model of dsDNA which is solely based on experimentally determined knotting probabilities. This model is then used to analyse the statistics of DNA knotting and determine typical knot sizes, motivated by the emergence of DNA nanopore sequencing technology. This analysis naturally precedes further experimental or theoretical investigations designed to address the problem of how to avoid or control knots in DNA nanopore sequencing devices. Employing the aforementioned coarse-grained model, the abundance and size of knots in chains exceeding half a million base pairs is studied for physiologically relevant salt concentrations of 0.15M NaCl. Although it is well known that knots are likely to form in long polymer chains, no quantitative estimation of DNA knotting probabilities is available for chains beyond ≈50,000 base pairs [20, 21, 22]. To estimate knotting probabilities for DNA chains of up to half a million base pairs, model parameters are chosen so that predicted knotting probabilities of short DNA chains match knotting probabilities from electrophoresis experiments: DNA is modelled as a semi-flexible chain of impermeable spherical beads of diameter *d*≈4.5nm, which corresponds to ≈13 base pairs (bp). An intrinsic stiffness controls the bending of the chain and the effective DNA diameter *d* subsumes excluded volume effects as well as screened electrostatic interactions [20]. The simplicity of the model is the key to deriving optimal model parameters and obtaining statistical estimates for chain lengths which are relevant in the context of future applications of nanopore sequencing technology. Mathematical details of the model, as well as the rationale for choosing particular values of the model parameters are discussed in the Methods section.

## Results

Intriguingly, the DNA model predicts a persistence length of ≈50nm in excellent agreement with experimental findings [23]. We stress that experimentally measured knotting probabilities of short dsDNA chains are the only input to our model (see the Methods section). This observation is non-trivial, as it implies that metric properties of DNA can be predicted from purely (non-metric) topological properties, which has never been demonstrated before. Hence, basing a simple real chain model with stiffness on knotting probabilities allows for the description of physical properties of the chain. The model is then employed to predict knotting probabilities of long dsDNA chains as well as typical sizes of DNA knots. This is to be contrasted with the approach in [20], which in addition to knotting probabilities also requires the persistence length of DNA as an input parameter. Although the dsDNA model in [20] is coarser, and introduces a sequence of impermeable cylinders to model DNA, both approaches derive similar values for the effective diameter of DNA. In comparison, simple random walk models of DNA which lack excluded volume interactions [24] tend to overestimate the occurrence of knots. E.g., our real chain model predicts that a chain of 150,000 base pairs is knotted in roughly 40% of all cases, whereas random walks of 500 segments (assuming a Kuhn length of 300 base pairs) exhibit knots in ≈80% of all cases if the same closure is applied.

**Fig 1:**
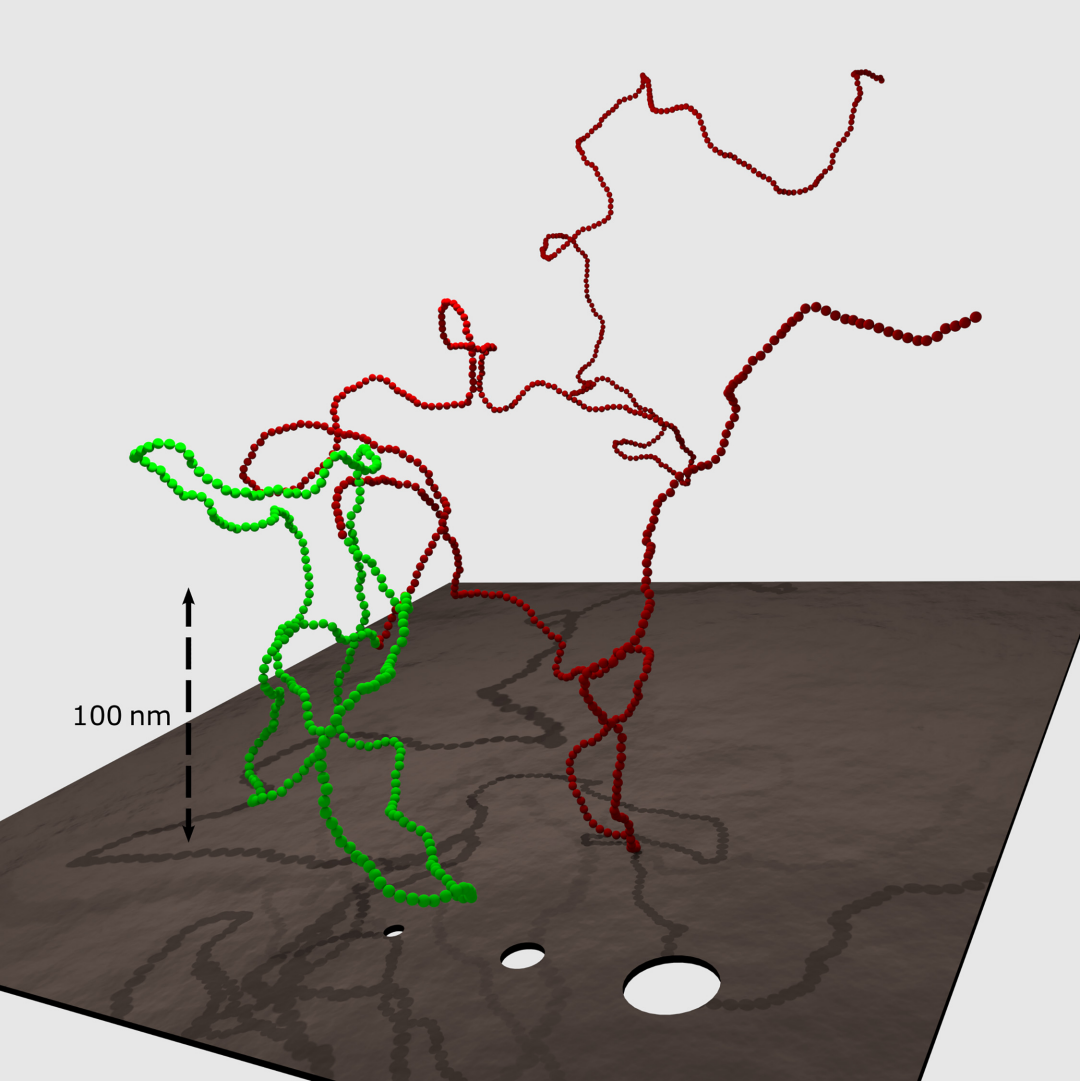
A typical DNA model chain with an embedded trefoil knot. The chain consists of 1,000 beads and corresponds to a double-stranded DNA molecule of ≈13,000 base pairs. This is roughly the size which can still be sequenced in current nanopore devices. The trefoil (light green) consists of ≈4,000 base pairs (N≈300) and has a diameter of ≈160nm. It is thus a typical representative of a knot in a DNA chain even if longer chains are considered. For comparison, the DNA chain is pictured in relation to nanopores with diameters of 5nm, 10nm and 20nm.

**Fig 2:**
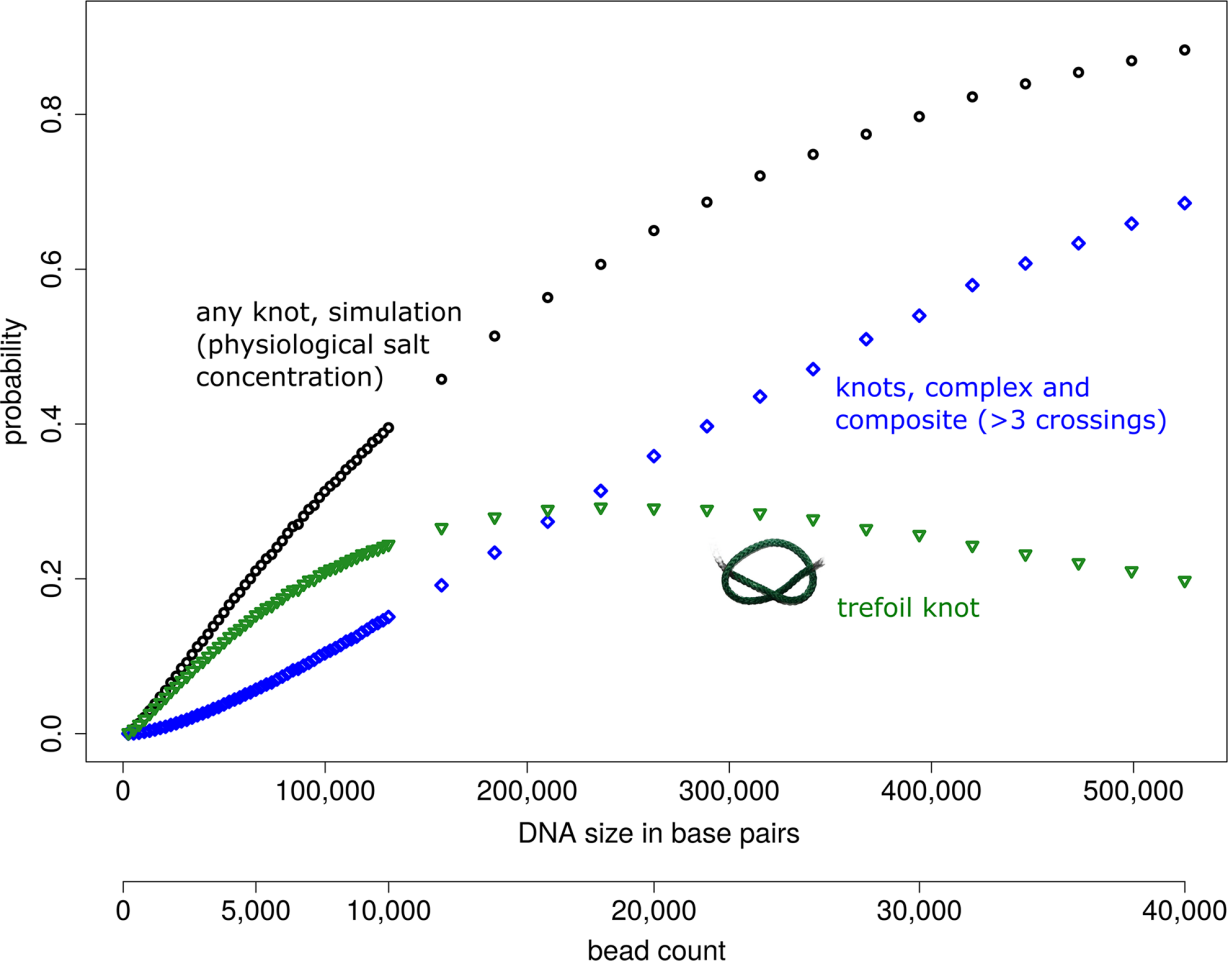
Probability of observing knots in dsDNA as a function of DNA length (in base pairs) for physiological salt concentrations (0.15M NaCl). The total knotting probability as well as the probability of trefoil knots are shown. The probability of complex and composite knots, i.e. knots with more than three crossings, is displayed to illustrate the increase of knot complexity with chain size. Error bars are smaller than symbol size. All data points and errors are documented in S1 Table. One bead of the coarse-grained chain corresponds to ≈13 base pairs.

**Fig 3:**
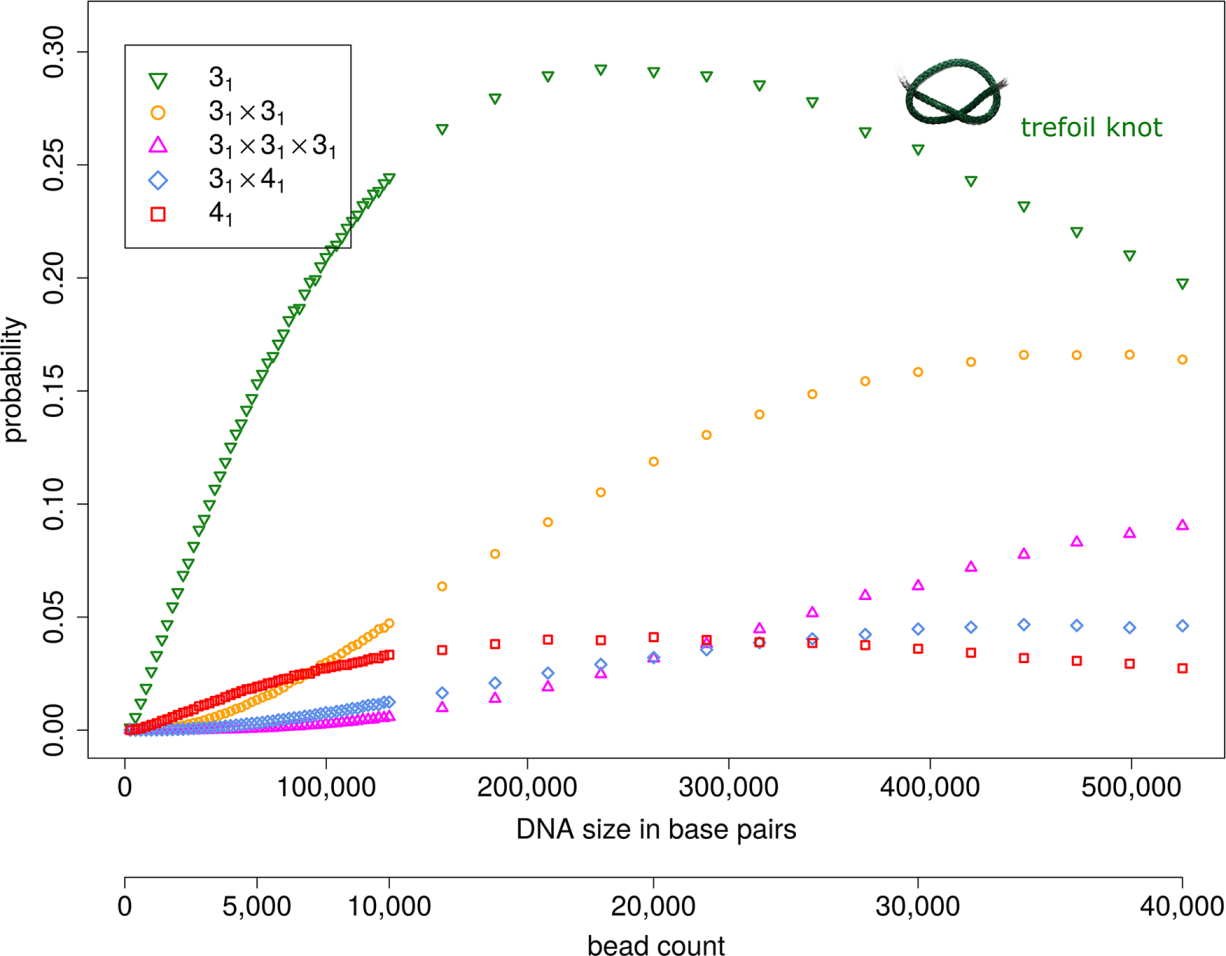
Probabilities of dominant knot types in dsDNA as a function of DNA length (in base pairs) for physiological salt concentrations (0.15M NaCl). The knot spectrum is dominated by composite knots which have trefoil knots as prime factors. The likelihood of observing a prime knot other than the trefoil 3_1_ or figure-eight knot 4_1_ is very small for DNA chains of up to ≈525,000bp (N = 40,000 beads), see S1 Fig. Data points are documented in S2 Table.

In Fig 1, a typical trefoil (light green) in a DNA chain of ≈13,000bp (represented by a coarse-grained model chain of N = 1,000 beads) is displayed in relation to characteristic nanopore sizes. In Fig 2 computed probabilities for observing knots under physiological salt conditions are shown for DNA strands of up to ≈525,000bp (N = 40,000, S1 Table, supporting information). At this length, ≈88% of all chains already contain at least one knot. Remarkably, more than ≈68% contain complex knots with more than three crossings or composite knots. The transition from a mostly unknotted to a mostly knotted ensemble of DNA chains is indicated by the base pair count *B*_0_ at which the probability of obtaining an unknotted conformation is 1/*e*≈0.37 [25]. *B*_0_≈250,000bp(N≈19,000 beads) also characterizes the regime where knots with higher crossing number (≥4) become more abundant than trefoil knots. Intriguingly, Fig 3 indicates that more complex entanglements are mainly made up of composite knots which include trefoil knots as prime factors. Beyond 300,000bp (N≈22,850) the triple trefoil knot and even the 3_1_#4_1_ composite knot occur more often than the figure-eight knot 4_1_, and formation of prime knots with more than four crossings is very unlikely (S1 Fig). Hence, probabilities of composite knots are not mere product probabilities of the constituent prime factors, reflecting the non-local structure of emerging polymer entanglements. Note that even though the Alexander polynomials of the analysed composite knots (Fig 3) are identical to the Alexander polynomials of specific prime knots with eight crossings (e.g. 3_1_#3_1_ and 8_20_ share the same polynomial), the influence of prime knots with eight crossings on observed knotting probabilities is expected to be negligible, since all prime knots with seven crossings already have vanishingly small probabilities.

**Fig 4:**
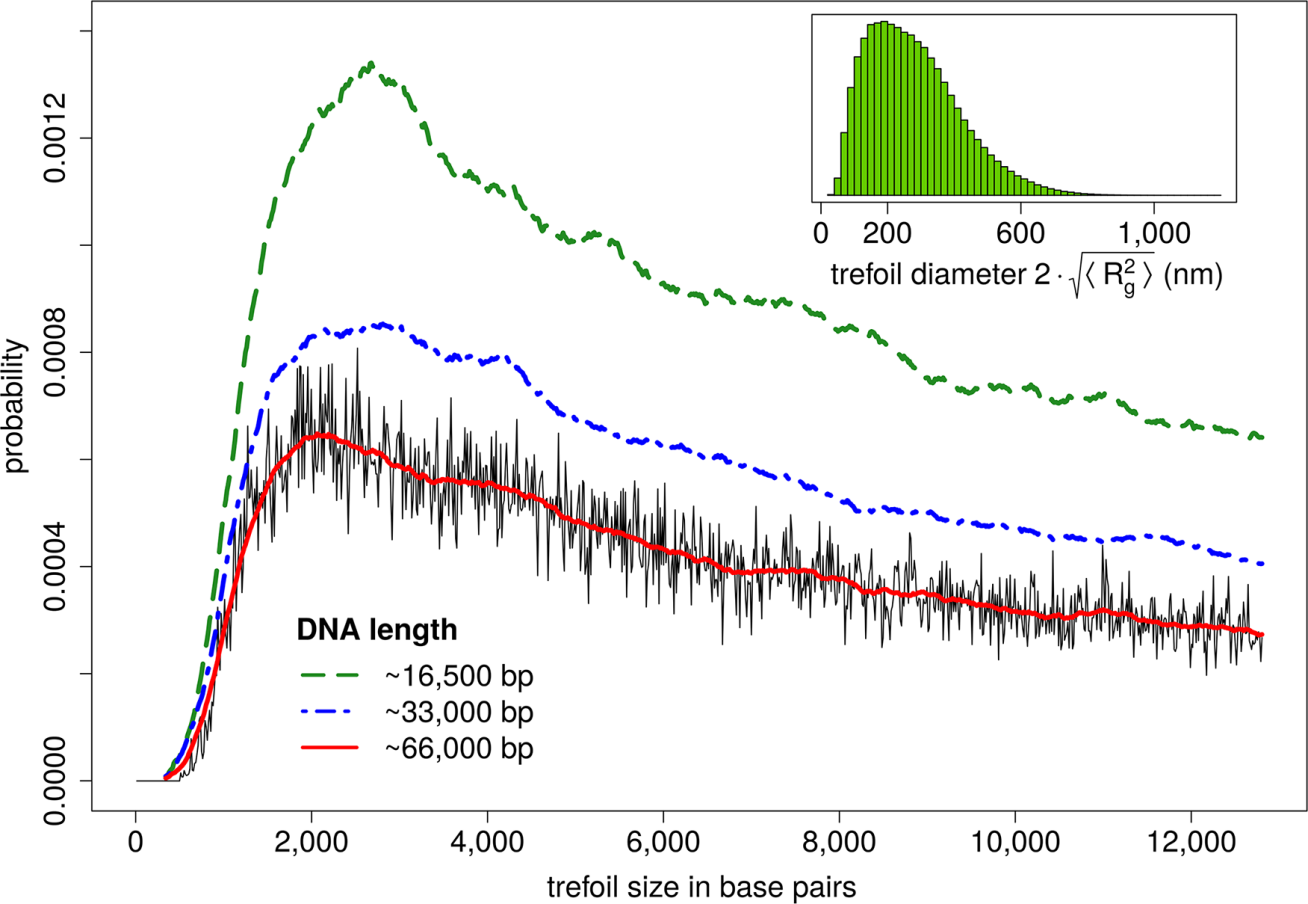
Probability distributions of the trefoil contour length for various DNA sizes. Displayed values are (conditional) probabilities of trefoil sizes of up to 13,000bp (N = 1000), given that a chain contains a trefoil knot in the first place. Therefore, the area below each individual curve equals one (if each curve is integrated over its entire domain). Irrespective of the actual DNA length, the most likely trefoil size is close to ≈3,000bp (N≈230). After reaching its maximum, each distribution decreases until the trefoil size is equal to the total DNA length. The original data has been smoothed by a (two-sided) running average. For comparison, the original data is shown for a total DNA length of ≈66,000bp (N = 5000). Inset: Distribution of trefoil diameters for a DNA strand of ≈13,000bp (N = 1000). The most likely diameter is ≈200nm.

In addition to estimating the mere abundance of knotted DNA chains, typical knot sizes can be determined as well: To identify the knotted region, a chain is trimmed from both ends until the remaining part becomes unknotted (see the Methods section). In Fig 4, the size of a trefoil knot refers to the contour length of the knotted region, and its distribution is shown for various DNA lengths. Intriguingly, the most likely size of a trefoil knot is around 3,000bp (N≈230), independent of strand size. This observation as well as computed distributions of trefoil contour lengths are in excellent agreement with recent simulation results [22]. In [22], typical knot sizes have been determined for a similar coarse-grained model, and for a range of model parameters which can be mapped onto dsDNA at various salt concentrations. Note that DNA models based on random walks predict smaller knots: The maximum of the size distribution is at around 7 segments, corresponding to 2100 base pairs [26]. As opposed to its most likely value, the expectation value of the knot size increases with DNA length (Fig 4). To estimate a trefoil's geometrical extent, the radius of gyration $\sqrt{\left\langle R_{g}^{2}\right\rangle }$ of the trefoil contour is computed, and its diameter is then taken to be $2\cdot \sqrt{\left\langle R_{g}^{2}\right\rangle }$. The inset in Fig 4 displays the distribution of trefoil diameters for a DNA chain of ≈13,000bp (N = 1000), and the most likely value is ≈200nm. The trefoil displayed in Fig 1 (light green) consists of ≈4,000 base pairs (N≈300) and has a diameter ≈160nm. It may thus be regarded as a typical representative, even if longer DNA chains are considered.

## Discussion

As our DNA model reproduces the correct persistence length of DNA by adjusting model parameters to match experimentally measured knotting probabilities of short dsDNA chains, it can be inferred that knots may be employed as a tool in polymer physics: Given sufficient experimental data on knot statistics of a particular polymer species, the parameters of any suitable polymer model may be chosen so that theoretical knotting probabilities match the experimental ones. As knot statistics are a means to quantify global topological properties of polymer chains, the resulting model is expected to thoroughly reproduce polymer entanglements. Whether such a procedure leads to a good imitation of polymer behaviour and physically consistent results depends, among other things, on the selection of a proper set of adjustable model parameters.

The successful derivation of the persistence length of dsDNA indicates that at least for physiological salt concentrations, our DNA model is capable of representing basic physical properties of DNA strands. Other salt concentrations will be tested in future investigations.

Simulations of this model indicate that DNA molecules beyond 250,000 base pairs are likely knotted and contain composite knots. Our analysis of knotting in dsDNA for physiologically relevant salt concentrations of 0.15M NaCl estimates lower bounds for DNA knotting in nanopore sequencing devices which adapt double-stranded DNA for sequencing: Most nanopore setups operate in high salt [27], which increases the likelihood of knotted conformations [20]. Furthermore, while most nanopore sequencing techniques keep DNA in its single-stranded form [28], very successful alternatives [29, 30] approach the problem by ligating adaptors to the ends of dsDNA, which subsequently help to thread the dsDNA into the pore, and afterwards control the translocation of a single DNA strand.

More specifically, it was demonstrated [31, 29] that DNA polymerase can slow down and control the transport of dsDNA through a biological nanopore: A polymerase molecule is anchored at the entrance of the nanopore, which splits the double-stranded DNA and drives the translocation of a single DNA strand. With this measure, sequence information can be obtained more reliably, at least for DNA strands of up to ≈4,500 base pairs [30].

If translocation is driven by DNA polymerase, every step in sequential movement takes several milliseconds [31]. This timespan should be long enough to equilibrate the knotted region or at least a substantial part of it, whereas diffusion along the contour of the DNA can probably be neglected [32]. The momentary size of a threaded trefoil can thus be estimated from ensemble statistics to be $2\cdot \sqrt{\left\langle R_{g}^{2}\right\rangle }$ (see the inset in Fig 4), and pulling a trefoil knot through the pore should not tighten it mechanically. However, knots with high crossing number or composite knots may behave differently, in which case $2\cdot \sqrt{\left\langle R_{g}^{2}\right\rangle }$ may not be a proper measure of knot size. If a knot is located close to the nanopore, the ion flux may be perturbed, and the magnitude of the perturbation is probably related to its geometrical extent.

Therefore, knotty problems may even occur for chain lengths which are within reach of current technology. The presence of complex and composite knots in long DNA chains might lead to a blockage of the nanopore's entrance. Though a blockage can potentially be avoided for simple knots as discussed in [4, 5], knots of any crossing number might significantly alter the ion transport along the pore or even the translocation dynamics of the DNA. As soon as knots become abundant in long DNA chains, interpretation of the current signal and discrimination of individual nucleotides may be prone to errors, even if DNA molecules can still be threaded through nanopores at reasonable rates, since the fingerprint of the DNA's nucleotide sequence sensitively depends on the resulting ion flux and DNA translocation dynamics. It is a very hard problem to ascertain how the time-dependent electrochemical current changes in the presence of a DNA knot. A quantitative analysis clearly goes beyond the scope of this paper.

We hope that our work will stimulate further experimental and theoretical investigations of the aforementioned issues. The vision that one day, a nanopore sequencing device could read a significant portion of a chromosome from just a single DNA molecule will have to include an idea of how to avoid knots in long DNA chains [33, 34].

## Methods

### Modelling and simulation of equilibrated single dsDNA strands

We employ a discrete worm-like chain (Kratky-Porod) model [35] with hard sphere interactions between beads and fixed bond lengths. The bending potential is given by

$$U/k_{B}T=-g\sum_{i}cos\;(\theta_{i})$$

with the *θ*_*i*_, *i* = 1,…,*N* – 1, being the angles between adjacent bond vectors. The computational model is fully determined in terms of the number of beads *N* and stiffness parameter *g* ≥ 0. Knotting probabilities of Kratky-Porod chains with excluded volume interactions depend on *g* in a non-trivial manner [36], whereas the knotting of ideal Kratky-Porod chains monotonously decreases with stiffness. Screened electrostatic interactions are absorbed in an effective hard sphere diameter *d*, which depends on the salt concentration [20]. In dsDNA the distance between adjacent base pairs is 0.34nm. A DNA strand of *B* base pairs is thus modelled as a chain of *N* = *B* · 0.34nm/*d* beads.

In previous experimental studies [20, 21], DNA knotting probabilities have been obtained by gel electrophoresis for dsDNA molecules with a length of 5.6kbp, 8.6kbp [21] and 10kbp [20] for different salt concentrations. Even though DNA had been cyclized before the knot type was determined, knots formed when DNA was still in a linear state. Therefore, experimental knotting probabilities are more likely to reflect probabilities in linear DNA. Either way, probabilities for knots in loops and knots in open chains are very similar, as has been demonstrated for random walks in [10] and for self-avoiding chains in [15]. DNA lengths from these experiments can be converted to chain lengths of the computational model for a given *d*. To obtain an optimal set of parameters (*g*,*d*) to model dsDNA under physiologically relevant salt concentrations of 0.15M NaCl, knotting probabilities are computed for 16 × 16 = 256 points of an equispaced grid with boundary points *N* = 250, 1,000, *g* = 6.5, 14. Each chain is simulated with Markov chain Monte Carlo (MCMC) methods, applying generalized MOS (inversion, reflection and interchange) [37], crank-shaft and pivot moves [38]. Typical MCMC errors are two orders of magnitude smaller than corresponding experimental errors [20, 21] and neglected in subsequent analysis. With a non-parametric regression in *R* [39] (library method *loess*), a surface is fitted to the grid of simulated knotting probabilities. The comparison of the interpolated knotting probabilities with the experimental results for a salt concentration of 0.15M NaCl defines a smooth (least squares) error function *E*(*g*,*d*), which is minimized with the aid of the Levenberg-Marquardt algorithm (*R* [39] library method *nls*.*lm*): The minimum (*g*,*d*) of the error function *E* is interpreted as an optimal parameter set, yielding *g*≈11.673 and *d*≈4.465. Even though the physical diameter *D* of dsDNA is only about 2nm, the effective diameter *d* also accounts for the influence of screened Coulomb interactions in addition to excluded volume. Thus, *d* is in general larger than *D* and would approach *D* for high salt concentrations (for which electrostatic interactions of dsDNA are completely screened) [20]. Production runs (Figs 2–4 and S1) employ this parameter set to predict knotting probabilities for dsDNA strands of up to ≈525,000 base pairs (computed knotting probabilities and MCMC errors are documented in S1 Table, S2 Table and S3 Table). For the (ideal) Kratky-Porod chain, the functional dependence of the persistence length *l*_*p*_(*g*,*d*) is given by *l*_*p*_(*g*,*d*) = −*d*/*ln* (*coth*(*g*) − 1/*g*) [40], yielding ≈49.85nm.

### Knot analysis

As a topological knot is necessarily a closed space curve, the open DNA chain has to be closed prior to knot detection. Here, we join the ends of the chain by first extending them away from the centre of mass, and then connecting them by the legs of a triangle, so that the additionally constructed line segments do not interfere with the original chain volume [41]. For each closed curve, the Alexander polynomial is evaluated and used to identify the knot type [10]. Note that in principle, the implementation of the closure may create additional entanglements and knots. In practice, this effect only plays a minor role when calculating ensemble averages. Different closures result in almost identical knotting probabilities as was demonstrated for random walks in [10] and for self-avoiding chains in [15]. To determine the knotted region of a trefoil knot as shown in Fig 4, a chain is first trimmed bead by bead from one end and subsequently analysed until the remaining part becomes unknotted. The same procedure is then applied starting from the other terminus. The remaining beads define the contour length of the knot, and its radius of gyration $\sqrt{\left\langle R_{g}^{2}\right\rangle }$ is computed to describe the knot’s physical extent as $2\cdot \sqrt{\left\langle R_{g}^{2}\right\rangle }$.

## Supporting Information

S1 Table Probability of observing knots in dsDNA (simulation results, salt concentration *c* = 0.15M NaCl). (PDF)

S2 Table Probability of observing prime knots in dsDNA (simulation results, salt concentration *c* = 0.15M NaCl). (PDF)

S3 Table Probability of observing composite knots in dsDNA (simulation results, salt concentration *c* = 0.15M NaCl). (PDF)

S1 Fig Probability of observing prime knots in dsDNA as a function of DNA length (in base pairs) for physiological salt concentrations (0.15M NaCl). (TIF)
