# Supplementary material for: A Monte Carlo Study of Knots in Long Double-Stranded DNA Chains
Source: PLoS Comput Biol. 2016 Sep 15;12(9):e1005029. doi: 10.1371/journal.pcbi.1005029 (PMC5025000; doi:10.1371/journal.pcbi.1005029)
Supplement: S1 Table — (PDF) [file pcbi.1005029.s001.pdf]

S1 Table. Probability of observing knots in dsDNA (simulation results, salt concentration $c = 0.15$M NaCl).

Number of beads N	Equivalent number of base pairs $B = B(N)$	Probability p to observe any knot	Probability p_t to observe trefoil knot	MCMC error of p
200	2,626	0.00116	0.00112	3.67E-05
400	5,253	0.00614	0.00574	8.54E-05
600	7,879	0.0132	0.0121	1.37E-04
800	10,506	0.0210	0.0186	1.89E-04
1,000	13,132	0.0298	0.0260	2.33E-04
1,200	15,759	0.0385	0.0331	2.83E-04
1,400	18,385	0.0474	0.0400	3.12E-04
1,600	21,012	0.0557	0.0468	3.48E-04
1,800	23,638	0.0659	0.0547	4.06E-04
2,000	26,265	0.0743	0.0610	4.47E-04
2,200	28,891	0.0844	0.0686	4.85E-04
2,400	31,518	0.0919	0.0740	5.06E-04
2,600	34,144	0.1022	0.0814	5.55E-04
2,800	36,771	0.1123	0.0886	5.87E-04
3,000	39,397	0.1195	0.0934	6.24E-04
3,200	42,024	0.1286	0.0999	6.59E-04
3,400	44,650	0.1386	0.1067	7.01E-04
3,600	47,276	0.1470	0.1126	7.33E-04
3,800	49,903	0.1564	0.1186	7.66E-04
4,000	52,529	0.1664	0.1253	7.85E-04
4,200	55,156	0.1752	0.1311	8.17E-04
4,400	57,782	0.1824	0.1356	8.37E-04
4,600	60,409	0.1923	0.1416	8.59E-04
4,800	63,035	0.2001	0.1468	8.98E-04
5,000	65,662	0.2102	0.1534	9.55E-04
5,200	68,288	0.2175	0.1575	9.92E-04
5,400	70,915	0.2261	0.1626	9.58E-04
5,600	73,541	0.231	0.165	1.01E-03
5,800	76,168	0.241	0.171	1.03E-03
6,000	78,794	0.249	0.175	1.07E-03
6,200	81,421	0.259	0.181	1.10E-03
6,400	84,047	0.267	0.186	1.15E-03
6,600	86,674	0.270	0.187	1.10E-03
6,800	89,300	0.281	0.193	1.17E-03
7,000	91,926	0.290	0.198	1.20E-03
7,200	94,553	0.295	0.199	1.22E-03
7,400	97,179	0.305	0.205	1.24E-03
7,600	99,806	0.313	0.209	1.25E-03

Number of beads N	Equivalent number of base pairs $B = B(N)$	Probability p to observe any knot	Probability p_t to observe trefoil knot	MCMC error of p
7,800	102,432	0.320	0.213	1.25E-03
8,000	105,059	0.325	0.215	1.28E-03
8,200	107,685	0.332	0.218	1.28E-03
8,400	110,312	0.341	0.222	1.35E-03
8,600	112,938	0.347	0.225	1.36E-03
8,800	115,565	0.353	0.228	1.33E-03
9,000	118,191	0.363	0.232	1.35E-03
9,200	120,818	0.368	0.234	1.39E-03
9,400	123,444	0.377	0.237	1.42E-03
9,600	126,071	0.381	0.238	1.39E-03
9,800	128,697	0.388	0.242	1.44E-03
10,000	131,324	0.395	0.244	1.46E-03
12,000	157,588	0.458	0.266	1.41E-03
14,000	183,853	0.514	0.280	1.46E-03
16,000	210,118	0.563	0.290	1.48E-03
18,000	236,382	0.606	0.293	1.50E-03
20,000	262,647	0.650	0.291	1.48E-03
22,000	288,912	0.687	0.290	1.69E-03
24,000	315,176	0.721	0.285	1.68E-03
26,000	341,441	0.748	0.277	1.61E-03
28,000	367,706	0.774	0.265	1.60E-03
30,000	393,971	0.797	0.257	1.53E-03
32,000	420,235	0.823	0.243	1.46E-03
34,000	446,500	0.839	0.232	1.40E-03
36,000	472,765	0.854	0.221	1.37E-03
38,000	499,029	0.869	0.210	1.31E-03
40,000	525,294	0.883	0.198	1.27E-03
