# Supplementary material for: A Monte Carlo Study of Knots in Long Double-Stranded DNA Chains
Source: PLoS Comput Biol. 2016 Sep 15;12(9):e1005029. doi: 10.1371/journal.pcbi.1005029 (PMC5025000; doi:10.1371/journal.pcbi.1005029)
Supplement: S2 Table — (PDF) [file pcbi.1005029.s002.pdf]

S2 Table. Probability of observing prime knots in dsDNA (simulation results, salt concentration $c = 0.15$M NaCl).

Number of beads N	Probability to observe prime knot 4_1	Probability to observe prime knot 5_1	Probability to observe prime knot 5_2	Probability to observe prime knot 6_1	Probability to observe prime knot 6_2	Probability to observe prime knot 6_3
200	0.00004	0.00000	0.00000	0.00000	0.00000	0.00000
400	0.00029	0.00004	0.00005	0.00000	0.00000	0.00000
600	0.0008	0.0001	0.0002	0.0000	0.0000	0.0000
800	0.0016	0.0002	0.0003	0.0000	0.0000	0.0000
1,000	0.0024	0.0003	0.0006	0.0001	0.0001	0.0000
1,200	0.0030	0.0006	0.0008	0.0001	0.0001	0.0001
1,400	0.0041	0.0007	0.0011	0.0002	0.0002	0.0001
1,600	0.0048	0.0008	0.0014	0.0002	0.0002	0.0001
1,800	0.0058	0.0011	0.0017	0.0003	0.0002	0.0001
2,000	0.0067	0.0012	0.0020	0.0003	0.0003	0.0002
2,200	0.0077	0.0014	0.0024	0.0004	0.0004	0.0002
2,400	0.0083	0.0016	0.0026	0.0005	0.0004	0.0002
2,600	0.0092	0.0018	0.0032	0.0005	0.0005	0.0003
2,800	0.0105	0.0022	0.0034	0.0006	0.0005	0.0003
3,000	0.0112	0.0023	0.0037	0.0006	0.0006	0.0004
3,200	0.0119	0.0025	0.0040	0.0007	0.0007	0.0004
3,400	0.0129	0.0028	0.0044	0.0007	0.0008	0.0004
3,600	0.0135	0.0028	0.0047	0.0008	0.0008	0.0005
3,800	0.0146	0.0031	0.0050	0.0009	0.0009	0.0005
4,000	0.0155	0.0032	0.0054	0.0010	0.0010	0.0005
4,200	0.0163	0.0035	0.0056	0.0010	0.0010	0.0006
4,400	0.0172	0.0036	0.0059	0.0011	0.0010	0.0006
4,600	0.0181	0.0038	0.0063	0.0011	0.0011	0.0006
4,800	0.0184	0.0040	0.0066	0.0012	0.0011	0.0006
5,000	0.0192	0.0041	0.0066	0.0013	0.0013	0.0007
5,200	0.0197	0.0044	0.0072	0.0013	0.0013	0.0007
5,400	0.0207	0.0043	0.0075	0.0013	0.0014	0.0008
5,600	0.021	0.005	0.008	0.001	0.001	0.001
5,800	0.022	0.005	0.008	0.001	0.001	0.001
6,000	0.023	0.005	0.009	0.002	0.002	0.001
6,200	0.023	0.005	0.009	0.002	0.002	0.001
6,400	0.024	0.005	0.009	0.002	0.002	0.001
6,600	0.025	0.005	0.009	0.002	0.002	0.001
6,800	0.025	0.005	0.009	0.002	0.002	0.001
7,000	0.025	0.006	0.010	0.002	0.002	0.001
7,200	0.026	0.006	0.010	0.002	0.002	0.001
7,400	0.027	0.006	0.010	0.002	0.002	0.001
7,600	0.027	0.006	0.010	0.002	0.002	0.001
7,800	0.028	0.006	0.010	0.002	0.002	0.001

Number of beads N	Probability to observe prime knot 4_1	Probability to observe prime knot 5_1	Probability to observe prime knot 5_2	Probability to observe prime knot 6_1	Probability to observe prime knot 6_2	Probability to observe prime knot 6_3
8,000	0.028	0.006	0.010	0.002	0.002	0.001
8,200	0.029	0.006	0.011	0.002	0.002	0.001
8,400	0.029	0.007	0.011	0.002	0.002	0.001
8,600	0.030	0.007	0.011	0.002	0.002	0.001
8,800	0.030	0.007	0.012	0.002	0.002	0.001
9,000	0.030	0.007	0.012	0.002	0.002	0.001
9,200	0.031	0.007	0.012	0.002	0.002	0.001
9,400	0.032	0.007	0.012	0.002	0.002	0.001
9,600	0.032	0.007	0.012	0.002	0.002	0.001
9,800	0.033	0.007	0.012	0.002	0.002	0.001
10,000	0.033	0.007	0.012	0.002	0.003	0.001
12,000	0.035	0.008	0.014	0.003	0.003	0.001
14,000	0.038	0.009	0.015	0.003	0.003	0.002
16,000	0.040	0.009	0.015	0.003	0.003	0.002
18,000	0.040	0.009	0.016	0.003	0.003	0.002
20,000	0.041	0.010	0.016	0.003	0.003	0.002
22,000	0.040	0.009	0.016	0.003	0.003	0.002
24,000	0.039	0.009	0.016	0.003	0.003	0.002
26,000	0.039	0.009	0.015	0.003	0.003	0.002
28,000	0.038	0.009	0.015	0.003	0.003	0.002
30,000	0.036	0.008	0.014	0.003	0.003	0.002
32,000	0.034	0.008	0.014	0.003	0.003	0.002
34,000	0.032	0.007	0.013	0.003	0.003	0.002
36,000	0.031	0.008	0.012	0.003	0.003	0.002
38,000	0.029	0.007	0.012	0.002	0.002	0.001
40,000	0.027	0.007	0.011	0.003	0.002	0.001
