# Supplementary material for: A Monte Carlo Study of Knots in Long Double-Stranded DNA Chains
Source: PLoS Comput Biol. 2016 Sep 15;12(9):e1005029. doi: 10.1371/journal.pcbi.1005029 (PMC5025000; doi:10.1371/journal.pcbi.1005029)
Supplement: S3 Table — (PDF) [file pcbi.1005029.s003.pdf]

S3 Table. Probability of observing composite knots in dsDNA (simulation results, salt concentration $c = 0.15$M NaCl).

Number of beads N	Probability to observe composite knot $3_1\#3_1$	Probability to observe composite knot $3_1\#3_1\#3_1$	Probability to observe composite knot $3_1\#4_1$
200	0.00000	0.00000	0.00000
400	0.00001	0.00000	0.00000
600	0.0000	0.0000	0.0000
800	0.0001	0.0000	0.0000
1,000	0.0002	0.0000	0.0000
1,200	0.0004	0.0000	0.0001
1,400	0.0007	0.0000	0.0001
1,600	0.0008	0.0000	0.0002
1,800	0.0012	0.0000	0.00031
2,000	0.0016	0.0000	0.0003
2,200	0.0021	0.0000	0.0004
2,400	0.0026	0.0001	0.0006
2,600	0.0032	0.0001	0.0007
2,800	0.0036	0.0001	0.0008
3,000	0.0043	0.0001	0.0010
3,200	0.0051	0.0002	0.0011
3,400	0.0058	0.0002	0.0013
3,600	0.0067	0.0003	0.0014
3,800	0.0074	0.0003	0.0018
4,000	0.0083	0.0003	0.0020
4,200	0.0094	0.0004	0.0022
4,400	0.0100	0.0005	0.0024
4,600	0.0112	0.0006	0.0027
4,800	0.0124	0.0006	0.0030
5,000	0.0134	0.0008	0.0032
5,200	0.0144	0.0008	0.0034
5,400	0.0153	0.0009	0.0038
5,600	0.016	0.001	0.004
5,800	0.018	0.001	0.005
6,000	0.019	0.001	0.005
6,200	0.021	0.002	0.005
6,400	0.022	0.002	0.005
6,600	0.023	0.002	0.006
6,800	0.024	0.002	0.006
7,000	0.026	0.002	0.006
7,200	0.026	0.002	0.007
7,400	0.029	0.002	0.007
7,600	0.030	0.003	0.008
7,800	0.031	0.003	0.008

Number of beads N	Probability to observe composite knot $3_1\#3_1$	Probability to observe composite knot $3_1\#3_1\#3_1$	Probability to observe composite knot $3_1\#4_1$
8,000	0.032	0.003	0.008
8,200	0.034	0.003	0.009
8,400	0.035	0.004	0.009
8,600	0.037	0.004	0.010
8,800	0.038	0.004	0.010
9,000	0.040	0.004	0.010
9200	0.041	0.005	0.011
9,400	0.043	0.005	0.011
9,600	0.045	0.005	0.011
9,800	0.045	0.005	0.012
10,000	0.047	0.006	0.012
12,000	0.064	0.010	0.016
14,000	0.078	0.014	0.021
16,000	0.092	0.019	0.025
18,000	0.105	0.025	0.029
20,000	0.119	0.032	0.032
22,000	0.131	0.038	0.036
24,000	0.140	0.045	0.039
26,000	0.149	0.052	0.040
28,000	0.154	0.059	0.042
30,000	0.158	0.064	0.045
32,000	0.163	0.072	0.046
34,000	0.166	0.078	0.047
36,000	0.166	0.083	0.046
38,000	0.166	0.087	0.045
40,000	0.164	0.090	0.046
