# Supplementary figures and images for: A Monte Carlo Study of Knots in Long Double-Stranded DNA Chains
Source: PLoS Comput Biol. 2016 Sep 15;12(9):e1005029. doi: 10.1371/journal.pcbi.1005029 (PMC5025000; doi:10.1371/journal.pcbi.1005029)

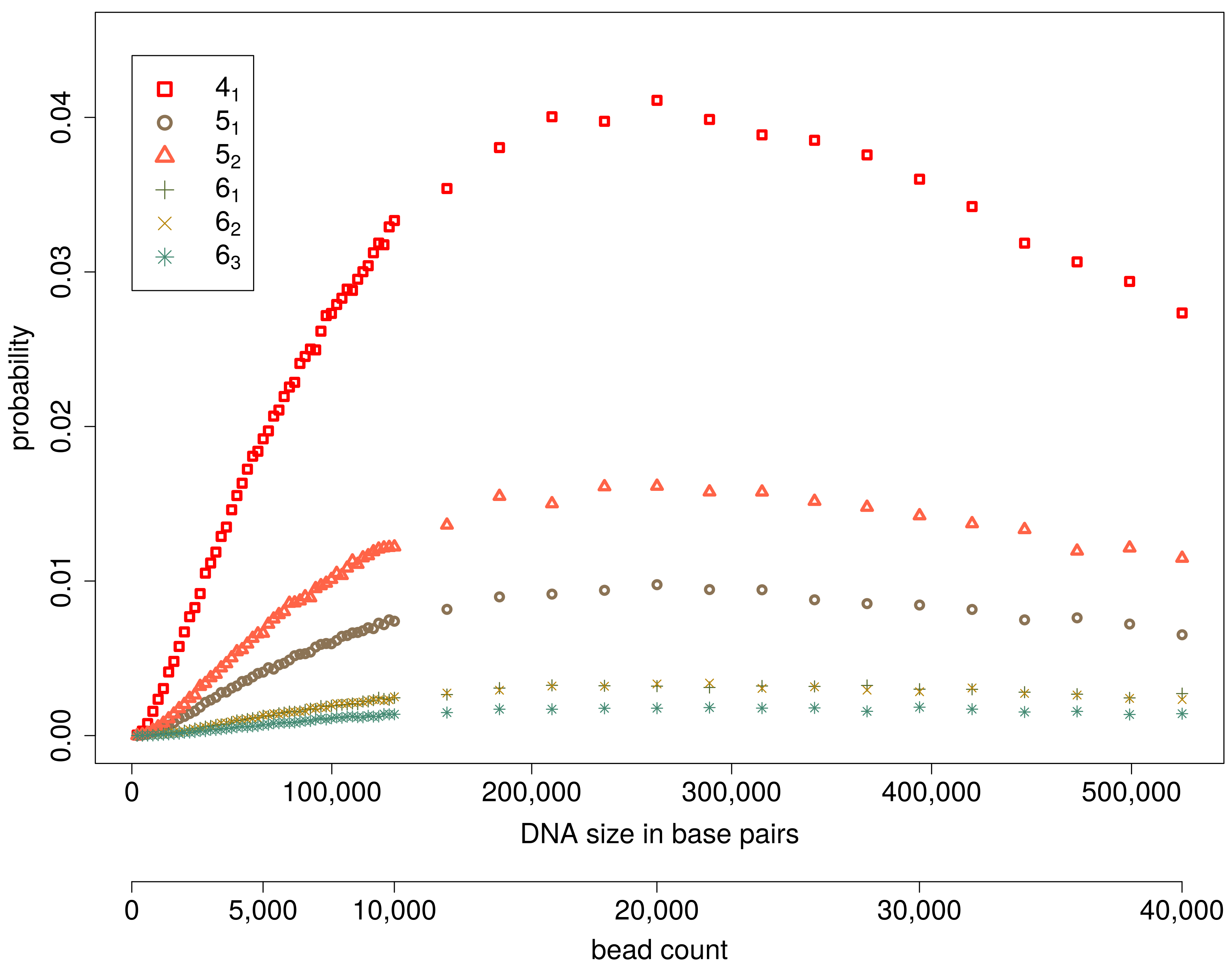

Supplement: S1 Fig — (TIF) [file pcbi.1005029.s004.tif]
